# Influence of Timing of Postoperative Weight-Bearing on Implant Failure Rate Among Older Patients With Intertrochanteric Hip Fractures: A Propensity Score Matching Cohort Study

**DOI:** 10.3389/fmed.2021.795595

**Published:** 2021-12-20

**Authors:** Xiaoyang Jia, Minfei Qiang, Kun Zhang, Qinghui Han, Ying Wu, Yanxi Chen

**Affiliations:** ^1^Department of Orthopedic Trauma, East Hospital, Tongji University School of Medicine, Shanghai, China; ^2^Department of Orthopedic Surgery, Zhongshan Hospital, Fudan University, Shanghai, China; ^3^Guangdong Provincial Key Laboratory of Tropical Disease Research, Department of Biostatistics, School of Public Health, Southern Medical University, Guangdong, China

**Keywords:** hip fracture (HFr), intertrochanteric fracture, timing of weight-bearing, immediate weight-bearing, restricted weight-bearing

## Abstract

**Introduction:** The purpose of this study was to determine whether immediate weight-bearing as tolerated increased the risk of implant failure and decreased functional outcomes compared with restricted weight-bearing.

**Methods:** From January 2010 to December 2018, 1,125 consecutive patients (≥65 years) with intertrochanteric fractures were identified. Of them, 130 patients were excluded, resulting in 995 patients in final cohort (563 receiving immediate weight-bearing and 432 receiving restricted weight-bearing). Propensity score (PS) matching yielded 403 patient pairs. Primary outcome was implant failure at 12 months. Secondary outcomes were implant failure at 3 months, functional outcomes at 12 months, and time to full weight-bearing.

**Results:** Among 806 patients who were matched by PS, the mean age was 77.8 years (SD, 7.6), and 603 patients (74.8%) were women. After matching, there was no significant difference between immediate (10.0% [39/389]) and restricted (9.1%, [35/385]) weight-bearing for implant failure at 12 months (absolute risk difference, 0.93% [95% CI, −3.26 to 5.13%]; RR, 1.11 [95% CI, 0.69 to 1.80]; *p* = 0.66). Additionally, no significant difference was seen for implant failure at 3 months and functional outcomes at 12 months. Patients with immediate weight-bearing had shorter time to full weight-bearing (mean [SD], 87.6 days [7.5] vs. 121.3 days [11.0]; mean difference, −33.7 [95% CI, −35.0 to −32.3]; *p* < 0.001).

**Conclusions:** Among older patients with intertrochanteric fractures, receipt of immediate weight-bearing as tolerated did not increase risks of implant failure or worsen functional outcomes compared with receipt of restricted weight-bearing. However, patients receiving immediate weight-bearing had a shorter time to full weight-bearing.

## Introduction

Annually, more than 1,500,000 adults worldwide suffer hip fracture (1–3). With the increasingly aging of the population, the annual number of hip fractures will be doubled by 2,040 (4–6), and intertrochanteric fractures account for nearly half of hip fractures (4). About 95% of intertrochanteric fractures are treated surgically in high-income countries (7), which aims to achieve early weight-bearing after surgery. Although postoperative early weight-bearing rehabilitation is strongly recommended by the American Academy of Orthopedic Surgeons guideline (8, 9), the appropriate timing of weight-bearing remains controversial.

Previous studies have shown that weight-bearing restrictions for patients for hip fractures are the risk factors for complications, such as venous thromboembolism, pneumonia, urinary tract infection, and pressure ulcers (10, 11). Weight-bearing recommendation under these circumstances may be for immediate weight-bearing as tolerated. However, appropriately a quarter of patients were still advised to receive restricted weight-bearing in the actual clinical setting (11, 12) due to that some surgeons perceive a lack of evidence-based guidelines based on high-quality researches on weight-bearing recommendations for hip fractures (13, 14). Moreover, a Cochrane review found insufficient evidence in the previous studies to establish the difference in the effectiveness between weight-bearing strategies for patients with hip fractures (15).

Weight-bearing restrictions for patients with lower extremity fractures may be attributed to a long-believed concern of fracture fixation failure if the osteosynthesis construct is loaded early (12). Although the failure of internal fixation is the largest risk of postoperative immediate weight-bearing, no study has yet evaluated this outcome (11). Therefore, the purpose of this retrospective cohort study was to determine whether immediate weight-bearing as tolerated was associated with the increase in the risks of postoperative complications and the decrease in the postoperative functional outcomes compared with restricted weight-bearing among older patients with intertrochanteric hip fractures. We hypothesized that patients with immediate weight-bearing as tolerated would have worse outcomes as compared to those with weight-bearing restrictions.

## Methods

### Data Sources

This retrospective cohort study was conducted using the Hospital Information System, a level 1 trauma center. The system contains patient data, including demographic characteristics at admission, injury details, and surgical records. The study was approved by our Institutional Review Board, with a waiver of informed consent because all data were deidentified.

### Study Population

Between January 1, 2010 and December 31, 2018, all patients aged 65 years or older on the date of admission, who had an intertrochanteric fracture and were treated with the proximal femoral nail antirotation 2 (PFNA-II) technique, were identified. Patients who had multiple traumatic injuries, pathological fractures, open reduction and internal fixation, bilateral intertrochanteric fractures, fractures that occurred as inpatient, previous fracture or surgery on the currently fractured site, cognitive disabilities preventing them from following the rehabilitation, disabilities of lower limbs before fracture, and transfer to another hospital after surgery or discharge against medical advice were excluded.

### Exposure Variable

The exposure was the type of weight-bearing received by patients with intertrochanteric fractures, which consisted of either immediate weight-bearing as tolerated or restricted weight-bearing. Patients were defined as receiving immediate weight-bearing as tolerated if they started weight-bearing within 7 days after surgery as tolerated. At first, the standing and walking were accomplished with the assistive device, and the limitation on weight-bearing only depended on their perception of pain or swelling at the fracture site. As the walking ability improved, the assistive device could be changed, and patients gradually achieved more weight-bearing when tolerated under the guidance of physical therapists. The purpose of the protocol was not to achieve full weight-bearing as soon as possible but to stimulate patients to increase weight-bearing according to their ability. Patients were defined as receiving restricted weight-bearing if they chose to receive 6 weeks of nonweight-bearing rehabilitation before starting weight-bearing exercise (16, 17). As we know, about 2–3 weeks after surgery, pain and swelling are reduced and soft callus is formed, which roughly corresponds to the time when the fragments are no longer moving freely. Then, the rehabilitation process of weight-bearing was the same as those in the immediate weight-bearing as a tolerated group.

All surgical procedures were implemented in line with the standard process and performed or supervised by a senior trauma surgeon with over 20 years of experience in treating hip fractures. All patients received comprehensive and interdisciplinary perioperative care, including the provision of venous thromboprophylaxis and antibiotic prophylaxis and the evaluation for and treatment of osteoporosis (9, 18).

### Outcomes

The primary outcome was the occurrence of implant failure (including hip varus deformity, screw cutout, stress fracture of femoral shaft, and nonunion) within 12 months after surgery. Because the rate of each complication was relatively low, a composite complication (implant failure) was defined as the primary outcome. Hip varus deformity was defined as the femoral neck shaft angle <120° (19). A fracture that had not healed for more than 8 months or had abnormal activity at the broken end was defined as nonunion (20). The secondary outcomes included the following: (1) implant failure (which did not include nonunion because of the nonunion definition) within 3 months; (2) the Lower Extremity Functional Scale (LEFS) at 12 months (21); (3) the Harris hip score at 12 months (22); (4) the visual analog scale (VAS) at 12 months; and (5) the time to full weight-bearing. The LEFS outcome is utilized to evaluate the function of lower limbs (score range, 0–80 points, with higher scores indicating a better activity). The Harris hip score provides a numerical rating of hip function (score range, 0–100 points, with 0–69 indicating poor function, 70–79 indicating fair function, 80–89 indicating good function, and 90–100 indicating excellent function). The VAS is a numerical rating scale of hip pain (score range, 0–10 points, with 0 indicating no pain and 10 indicating maximum pain). Implant failure was evaluated with anteroposterior and lateral radiographs collected during the follow-up period. Radiographs were assessed by two experienced orthopedic surgeons. Routine follow-up was at 1, 3, and 12 months after surgery in the outpatient department. When patients could not attend the outpatient department, the outcomes were collected by telephone or mail.

Any signs or symptoms of implant failure and functional outcomes were derived from the medical or follow-up record that was reviewed by an independent researcher who was blind to the type of weight-bearing. The follow-up period was from the day after the surgery to the end of the study period (December 31, 2019) or death, whichever occurred first.

### Statistical Analysis

Baseline patient characteristics were expressed as means with SD or median and interquartile range (IQR) for continuous variables and numbers and percentages for categorical variables.

To adjust for the potential confounding, the PS matching analysis was used. The predicted probability from the PS model was adjusted for likely confounders identified by a directed acyclic graph (Supplementary Figure S1) (23), including age, sex, body mass index, geographic region, education level, American Society of Anesthesiologists (ASA) level, injury mechanism, time from injury to operation, Singh index (24), AO Foundation/Orthopedic Trauma Association fracture classification (25), fracture reduction quality, type of anesthesia, drinking status, smoking status, drug use, and medical history were used to calculate the PS. One: one nearest-neighbor caliper matching was used to match patients based on the logit PS with a caliper of 0.02 of the SD of logit PS (26). For given covariates, a standardized difference of <10% indicates a relatively small imbalance (27, 28). Primary and secondary outcomes were compared between the 2 groups after matching. A logistic regression model with relative risk (RR) was used to compare patients with immediate weight-bearing as tolerated and restricted weight-bearing for implant failure in the PS-matched cohort, and a linear regression model for postoperative functional outcomes and time to full weight-bearing. The cumulative incidence of implant failure was calculated using the Kaplan–Meier method, and the log-rank test was used to calculate the *p-*value.

Prespecified subgroup analysis for primary outcome was performed stratified by age (65–74 or ≥ 75 years), sex, ASA level (1–2, 3, or 4), time from injury to operation ( ≤ 48 or > 48 h), Singh index (1, 2, 3, or 4–6), AO/OTA fracture classification (A1, A2, or A3), and reduction quality (good, acceptable, or poor). Whether RR was the same across the subgroups that were tested by the significance of the interaction terms. We performed 4 sensitivity analyses to assess the robustness of our study findings. First, because some patients who lost to follow-up were not included in the analysis of primary analysis, we performed multiple imputations to evaluate sensitivity to missing data under the assumption the data were missing at random. Missing values of the primary outcome were imputed by the chained equation method using all covariates as predictors. Point and interval estimates were obtained using Rubin's rules to combine the imputed observation (29). The primary outcome between the two groups after multiple imputations was compared using the same analysis model. Second, we repeated the primary analysis in the entire cohort using stabilized inverse probability of treating weighting (IPTW). Third, to explore the potential statistical effect of unmeasured confounders related to patient frailty on the study findings, we conducted a sensitivity analysis, using the multivariable logistic regression model with adjustment for the same covariates with PS matching for primary outcome on younger patients, aged 18–64 years. Fourth, we calculated the *E*-value to evaluate the influence of possible unmeasured and residual confounding factors on the primary outcome (i.e., the minimum strength of association of unmeasured confounders on exposure and outcome) to fully explain away the estimated exposure outcome association of interest (Supplementary Methods and Supplementary Table S1) (30).

Since multiple comparisons may lead to type I error, the results of the analyses of the secondary outcomes should be interpreted as exploratory. Two-sided overall values of *p* < 0.05 were considered statistically significant. All statistical analyses were performed using SAS software (version 9.4; SAS Institute Inc). An independent statistician unaware of the group assignment performed all the analyses.

## Results

### Study Population

**Figure 1:**
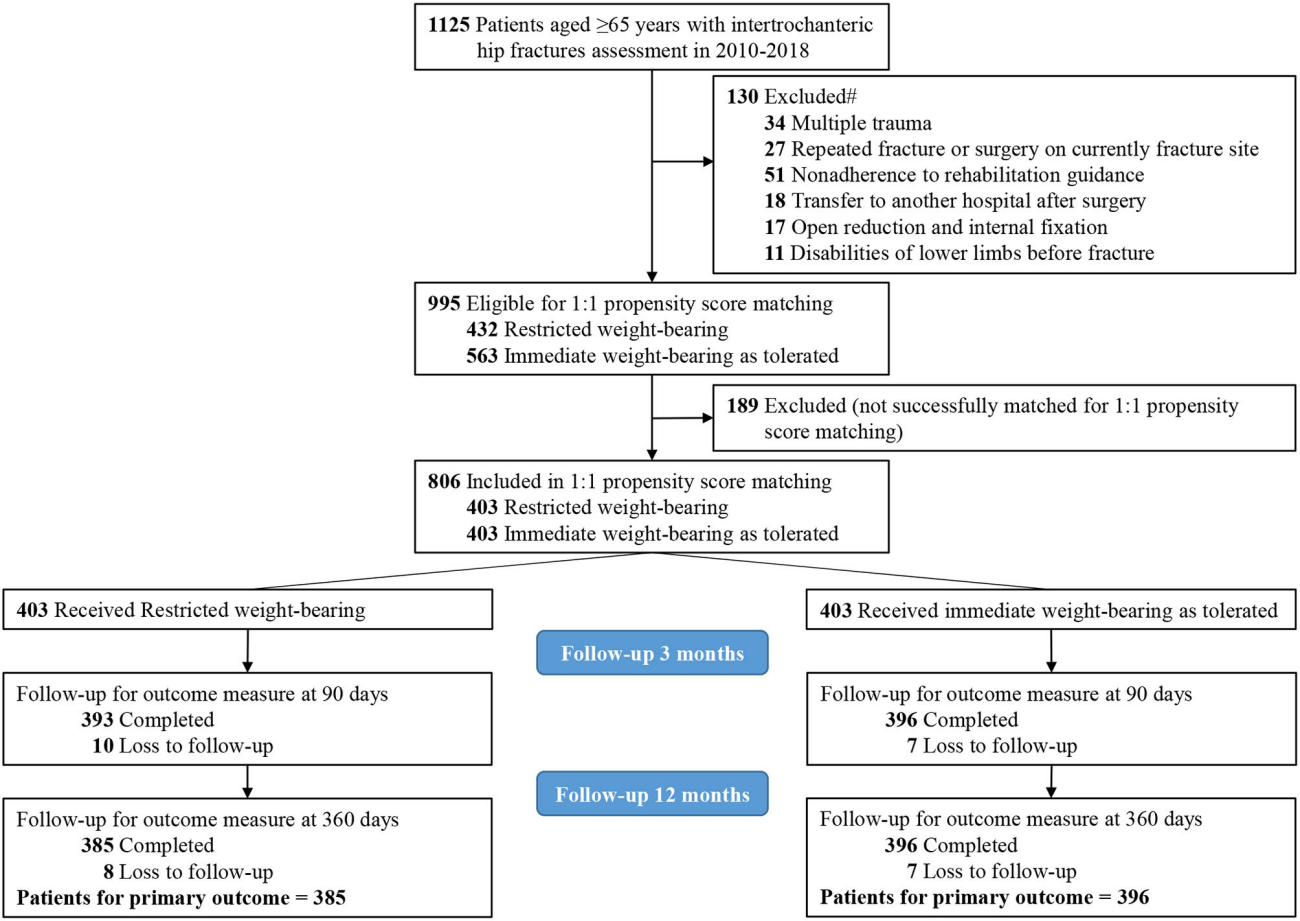
Flow diagram of eligible patients. #Each patient may be included in more than 1 exclusion group.

A total of 1,125 patients aged 65 years and older with intertrochanteric hip fractures were identified. Of those, 130 patients (11.6%) were excluded with 1 or more of the following criteria: repeated fracture or surgery on the currently fractured site (27 patients), nonadherence to postoperative rehabilitation guidance (51 patients), multiple trauma (34 patients), open reduction and internal fixation (17 patients), disabilities of lower limbs before fracture (11 patients), or transfer to another hospital after surgery (18 patients). The final study cohort of 995 patients included 563 patients (56.6%) who received immediate weight-bearing as tolerated and 432 patients (43.4%) who received restricted weight-bearing (Figure 1).

### Patient Characteristics

Among 995 patients who underwent hip surgery, the mean (SD) age was 77.8 (8.2) years, and 737 patients (74.1%) were women. Variables that differed between patients with restricted weight-bearing and immediate weight-bearing as tolerated included sex, body mass index (calculated as weight in kilograms divided by height in meters squared), geographic region, education level, ASA level, etc. (Table 1). Patients with restricted weight-bearing (*n* = 432 [43.4%]) compared with those with immediate weight-bearing as tolerated (*n* = 563 [56.6%]) were less likely to have a history of chronic kidney disease (21 patients [4.9%] vs. 41 patients [7.3%]) and were more likely to have a history of alcoholism (52 patients [12.0%] vs. 48 patients [8.5%]), chronic obstructive pulmonary disease (38 patients [8.8%] vs. 33 patients [5.9%]), and smoking (120 patients [27.8%] vs. 125 patients [22.2%]) (Table 1).

**Table 1:** Baseline demographic characteristics of patients before and after propensity score (PS) matching based on the type of weight-bearing.

	Before propensity score matching^*^			After propensity score matching^*^		
**Demographics**	**Restricted weight-bearing (*n* = 432)**	**Immediate weight-bearing (*n* = 563)**	**SMD, %**	**Restricted weight-bearing (*n* = 403)**	**Immediate weight-bearing (*n* = 403)**	**SMD, %**
**Age, mean (SD), y**	78.2 (8.4)	77.3 (7.9)	11.0	78.0 (7.6)	77.6 (7.6)	5.3
65–74	139 (32.2)	175 (31.1)	−2.3	128 (31.8)	120 (29.8)	−4.3
≥75	293 (67.8)	388 (68.9)	2.3	275 (68.2)	283 (70.2)	4.3
**Female**	332 (76.9)	405 (71.9)	−11.3	309 (76.7)	294 (73.0)	−8.5
**Body mass index^#^**						
<20	125 (28.9)	132 (23.5)	−12.5	115 (28.5)	103 (25.6)	−6.7
20–25	172 (39.8)	227 (40.3)	1.0	154 (38.2)	150 (37.2)	−2.0
>25	135 (31.3)	204 (36.2)	10.6	134 (33.3)	150 (37.2)	8.3
**Geographic region**						
Coastland	294 (68.1)	416 (73.9)	12.9	284 (70.5)	297 (73.7)	7.1
Inland	138 (31.9)	147 (26.1)	−12.9	119 (29.5)	106 (26.3)	−7.1
**Education level**						
Primary school	257 (59.5)	302 (53.6)	−11.6	238 (59.1)	228 (56.6)	−5.0
Junior high school	53 (12.3)	99 (17.6)	15.0	50 (12.4)	60 (14.9)	7.2
Senior high school or above	122 (28.2)	162 (28.8)	1.2	115 (28.5)	115 (28.5)	0
**ASA classification^§^**						
1–2	269 (62.3)	321 (57.0)	−10.8	249 (61.8)	242 (60.0)	−3.6
3	111 (25.7)	172 (30.6)	10.7	106 (26.3)	118 (29.3)	6.7
4	52 (12.0)	70 (12.4)	1.2	48 (11.9)	43 (10.7)	−3.9
**Injury mechanism**						
Falling from height	314 (72.7)	392 (69.6)	−6.6	293 (72.7)	289 (71.7)	−2.2
Traffic accident	62 (14.4)	98 (17.4)	8.2	55 (13.7)	63 (15.6)	5.6
Other	56 (13.0)	73 (13.0)	0.3	55 (13.7)	51 (12.7)	−2.9
**Time from injury to operation, h**						
≤ 48	185 (42.8)	256 (45.5)	5.3	179 (44.4)	180 (44.7)	0.5
>48	247 (57.2)	307 (54.5)	−5.3	224 (55.6)	223 (55.3)	−0.5
**Singh index-osteoporosis^¶^**						
1–2	96 (22.2)	121 (21.5)	−1.8	91 (22.6)	82 (20.4)	−5.4
3	139 (32.2)	187 (33.2)	2.2	129 (32.0)	145 (36.0)	8.4
4–6	197 (45.6)	255 (45.3)	−0.6	183 (45.4)	176 (43.7)	−3.5
**AO/OTA classification^†^**						
A1	205 (47.5)	300 (53.3)	11.6	198 (49.1)	210 (52.1)	6.0
A2	127 (29.4)	147 (26.1)	−7.4	116 (28.8)	104 (25.8)	−6.7
A3	100 (23.3)	116 (20.6)	−6.1	89 (22.1)	89 (22.1)	0
**Reduction quality of fracture**						
Good	231 (53.5)	305 (54.2)	1.4	214 (53.1)	217 (53.9)	1.5
Acceptable	104 (24.1)	145 (25.8)	3.9	95 (23.6)	105 (26.1)	5.7
Poor	97 (22.5)	113 (20.1)	−5.9	94 (23.3)	81 (20.1)	−7.8
**Type of anesthesia**						
General	411 (95.1)	532 (94.5)	−2.9	384 (95.3)	383 (95.0)	−1.1
Spinal or epidural	21 (4.9)	31 (5.5)	2.9	19 (4.7)	20 (5.0)	1.1
**Alcoholism**	52 (12.0)	48 (8.5)	−11.6	44 (10.9)	37 (9.2)	−5.7
**Current smoker**	143 (33.1)	125 (22.2)	−24.6	116 (28.8)	103 (25.6)	−7.3
**Aspirin and/or clopidogrel use**	56 (13.0)	86 (15.3)	6.7	54 (13.4)	62 (15.4)	5.7
**Calcium and/or vitamin D use**	85 (19.7)	96 (17.1)	−6.8	77 (19.1)	72 (17.9)	−3.2
**Medical history**						
Chronic kidney disease	21 (4.9)	41 (7.3)	10.2	20 (5.0)	25 (6.2)	5.4
COPD	38 (8.8)	33 (5.9)	−11.3	28 (7.0)	25 (6.2)	−2.9
Diabetes	108 (25.0)	139 (24.7)	−0.7	101 (25.1)	105 (26.1)	2.3
Hypertension	305 (70.6)	395 (70.2)	−1.0	283 (70.2)	288 (71.5)	2.7

*SMD, standardized mean difference; ASA, American Society of Anesthesiologists; AO/OTA, AO Foundation/Orthopedic Trauma Association; COPD, chronic obstructive pulmonary disease*.

* *Data are expressed as number (percentage) of patients unless otherwise indicated; Percentages may not total 100 because of rounding*.

# *The body mass index is the weight in kilogram divided by the square of the height in meters*.

§ *Range, 1–6; higher level indicates greater risk during anesthesia. Classifications include 1 (a healthy patient with no disease), 2 (a patient with mild systemic disease), 3 (a patient with severe systemic disease), 4 (a patient with severe systemic disease i.e., life-threatening), 5 (a patient who is not expected to survive with surgery), and 6 (a patient in whom brain death has occurred)*.

¶ *Range, 1–6; lower level indicates more severe osteoporosis. Grade 1 (even the principal compressive trabeculae are markedly reduced in number and are no longer prominent), Grade 2 (only the principal compressive trabeculae stand out prominently; the others have been resorbed more or less completely), Grade 3 (there is a break in the continuity of the principal tensile trabeculae opposite the greater trochanter; this grade indicates definite osteoporosis), Grade 4 (principal tensile trabeculae are markedly reduced in number but can still be traced from the lateral cortex to the upper part of the femoral neck), Grade 5 (the structure of principal tensile and principal compressive trabeculae is accentuated. Ward's triangle appears prominent), and Grade 6 (all the normal trabecular groups are visible, and the upper end of the femur seems to be completely occupied by cancellous bone)*.

† *Range, A1–A3; different classifications indicate different types of fracture. A1 (simple fracture), A2 (comminuted fracture involving the lateral cortex), and A3 (reverse oblique fracture)*.

Propensity score matching produced 403 patient pairs (mean [SD] age, 77.8 [7.6] years; 603 patients [74.8%] were female) followed up for a median (IQR) of 15 months (13–18) with maximum follow-up of 28 months. After matching, all characteristics between the two groups were balanced, with all standardized differences <10% (Table 1 and Supplementary Figure S2).

### Primary Outcomes

**Figure 2:**
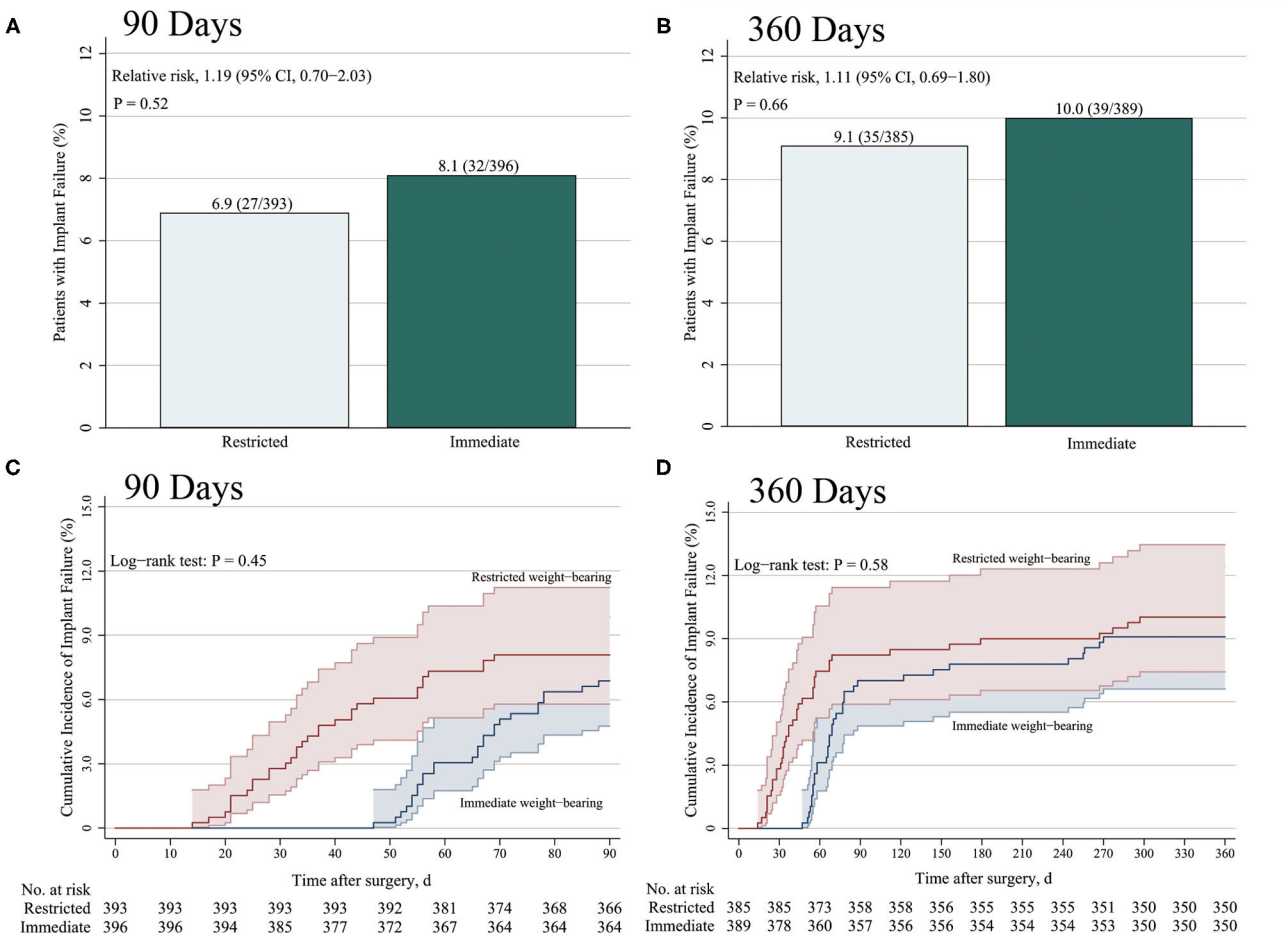
Incidence of implant failure at 3 months and 12 months after surgery. The primary end points were the cumulative incidence of implant failure at 3 months **(A)** and 12 months **(B)**. Kaplan–Meier estimates of the incidence of implant failure at 3 months **(C)** and 12 months **(D)** were shown. **(A,B)** *p-*values were calculated by means of the logistic regression model in the propensity score (PS)-matched cohort, **(C,D)** and by means of the Kaplan–Meier method using log-rank test.

Among 806 patients (403 patients in each group) in the matched cohort, 17 patients (2.1%; 10 patients with restricted weight-bearing and seven patients with immediate weight-bearing as tolerated) lost to follow-up within the 3 months after surgery, and 32 patients (4.0%; 18 patients with restricted weight-bearing and 14 patients with immediate weight-bearing as tolerated) lost to follow-up within the 12 months (Figure 1). After matching, there was no significant difference between restricted and immediate weight-bearing for implant failure at 12-month follow-up (restricted, 9.1%, [35/385 patients] vs. immediate, 10.0% [39/389 patients]; absolute risk difference, 0.93% [95% CI, −3.26% to 5.13%]; RR, 1.11 [95% CI, 0.69 to 1.80]; *p* = 0.66) (Table 2 and Figures 2B,D).

**Table 2:** Comparison of primary and secondary outcomes between PS-matched patients^*^.

**Outcome**	**Restricted weight-bearing** **(*****n*** **=** **403)^#^**		**Immediate weight-bearing** **(*****n*** **=** **403)**^*****^#^*****^		**Absolute risk difference or mean group-between difference (95% CI)**	**Relative risk (95% CI)**	***p*-value**
	**No. of patient**		**No. of patient**		**percentage points**	**percentage points**	
**Primary outcome**							
Implant failure at 12 months^§^	385	35 (9.1)	389	39 (10.0)	0.93 (−3.26 to 5.13)	1.11 (0.69 to 1.80)	0.66
Varus deformity	385	11 (2.9)	389	12 (3.1)	NA	NA	NA
Screw cutout	385	13 (3.4)	389	13 (3.3)	NA	NA	NA
Stress fracture of femoral shaft	385	6 (1.6)	389	10 (2.6)	NA	NA	NA
Nonunion	385	5 (1.3)	389	4 (1.0)	NA	NA	NA
**Secondary outcome**							
Categorical							
Implant failure at 3 months^§^	393	27 (6.9)	396	32 (8.1)	1.21 (−2.52 to 4.96)	1.19 (0.70 to 2.03)	0.52
Varus deformity	393	10 (2.5)	396	11 (2.8)	NA	NA	NA
Screw cutout	393	11 (2.8)	396	13 (3.3)	NA	NA	NA
Stress fracture of femoral shaft	393	6 (1.5)	396	8 (2.0)	NA	NA	NA
Nonunion^¶^	NA	NA	NA	NA	NA	NA	NA
Continuous							
LEFS score at 12 months^†^	385	64.6 (4.4)	389	64.9 (4.4)	0.32 (−0.30 to 0.94)	NA	0.31
Harris score at 12 months^‡^	385	79.2 (4.5)	389	78.8 (4.1)	−0.40 (−1.01 to 0.21)	NA	0.20
VAS score at 12 months^Φ^	385	1.5 (1.0)	389	1.5 (0.8)	−0.05 (−0.18 to 0.08)	NA	0.45
Time to full weight-bearing, d	385	121.3 (11.0)	389	87.6 (7.5)	−33.7 (−35.0 to −32.3)	NA	<0.001

*CI, confidence intervals; NA, not available; LEFS, Lower Extremity Functional Scale; VAS, visual analog scale*.

* *For implant failure, the absolute risk difference and relative risk (RR) are presented; for continuous outcomes, the mean group-between difference is presented. The p-values for implant failure are estimated with the use of the logistic regression analysis with the type of weight-bearing as a covariate; for continuous outcomes, the p-values are based on independent-sample t-test*.

# *Data for implant failure are expressed as number (percentage) of patients; data for continuous outcomes are expressed as mean (standard deviation) of patients*.

§ *Some patients experienced more than 1 implant failure. However, only the first implant failure was counted for each patient (e.g., a patient with varus deformity followed by screw cutout would be counted only as having a varus deformity in the category for varus deformity)*.

¶ *Nonunion is defined as the time of nonunion exceeding 8 months. Therefore, the outcome of nonunion is not included in the implant failure at 3 months*.

† *Range, 0 to 80; higher score indicates a better activity*.

‡ *Range, 0 to 100; higher score indicates a better function*.

Φ *Range, 0 to 10; higher score indicates a greater intensity of pain*.

### Secondary Outcomes

**Figure 3:**
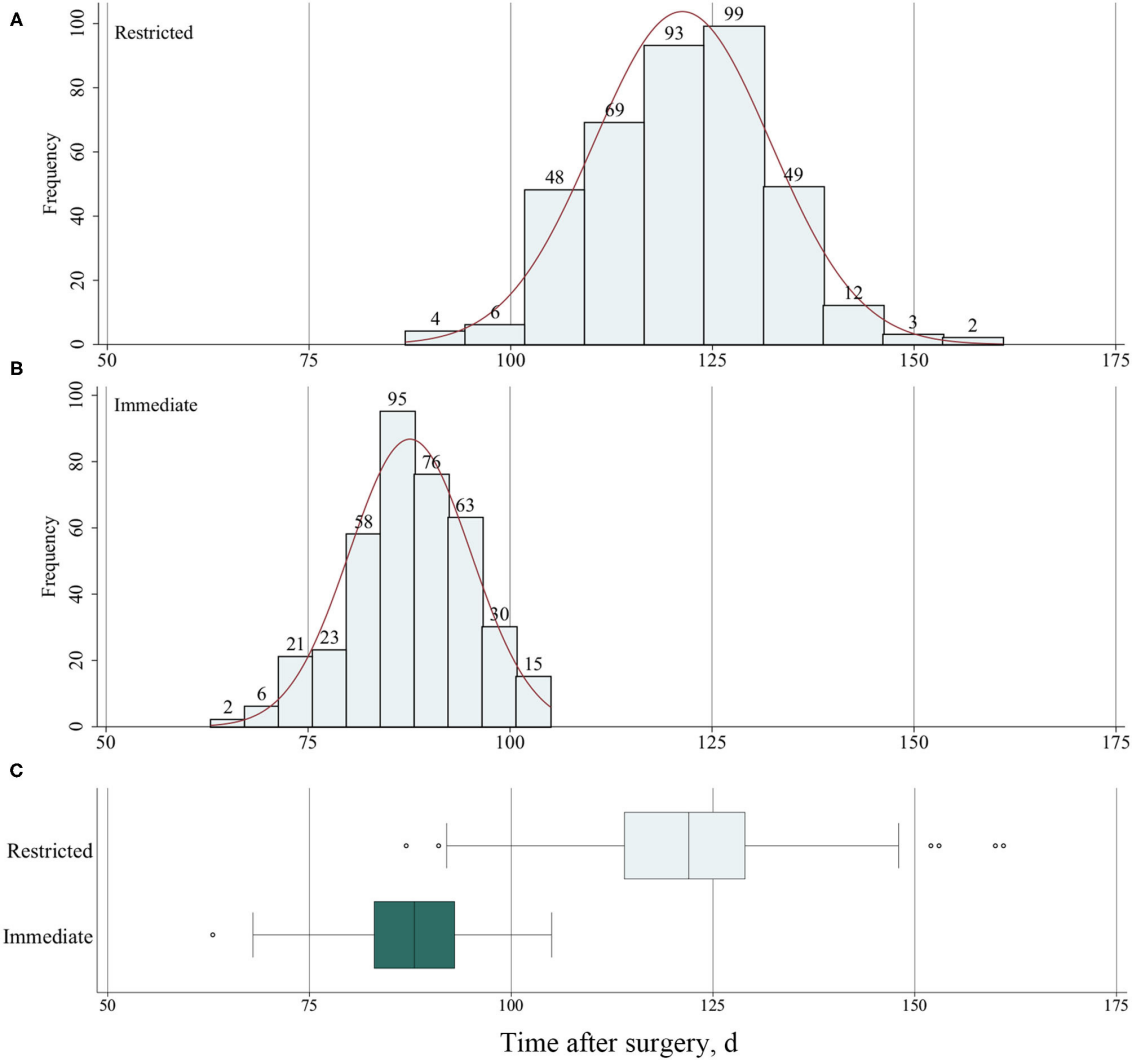
**(A)** Frequency of time to full weight-bearing for patients with restricted weight-bearing and **(B)** for patients with immediate weight-bearing as tolerated. **(C)** The time to full weight-bearing after surgery for patients with restricted and immediate weight-bearing.

Among 806 patients matched by PS, there was no difference between restricted and immediate weight-bearing for 3-month implant failure (restricted, 6.9%, [27/393 patients] vs. immediate, 8.1% [32/396 patients]; absolute risk difference, 1.21% [95% CI, −2.52% to 4.96%]; RR, 1.19 [95% CI, 0.70 to 2.03]; *p* = 0.52) (Table 2 and Figures 2A,C). At 12-month follow-up, there was no significant difference between two groups for the postoperative functional outcomes (Table 2). The mean score for LEFS was 64.6 points (SD, 4.1) in the restricted weight-bearing group vs. 64.9 points (SD, 4.4) in the immediate weight-bearing group (mean group-between difference, 0.32 points [95% CI, −0.30 to 0.94]; *p* = 0.31). The mean score for Harris was 79.2 points (SD, 4.5) in the restricted weight-bearing group vs. 78.8 points (SD, 4.1) in the immediate weight-bearing group (mean group-between difference, −0.40 [95% CI, −1.01 to 0.21]; *p* = 0.20). The mean score for VAS pain on affected side was 1.5 points (SD, 1.0) in the restricted weight-bearing group vs. 1.5 points (SD, 0.8) in the immediate weight-bearing group (mean group-between difference,−0.05 points [95% CI, −0.18 to 0.08]; *p* = 0.45) (Table 2). However, patients with immediate weight-bearing were associated with a shorter time to full weight-bearing (mean [SD], 87.6 days [7.5] vs. 121.3 days [11.0]; mean group-between difference, −33.7 [95% CI, −35.0 to −32.3]; *p* < 0.001) compared with those with restricted weight-bearing (Figure 3).

### Subgroup and Sensitivity Analyses

**Figure 4:**
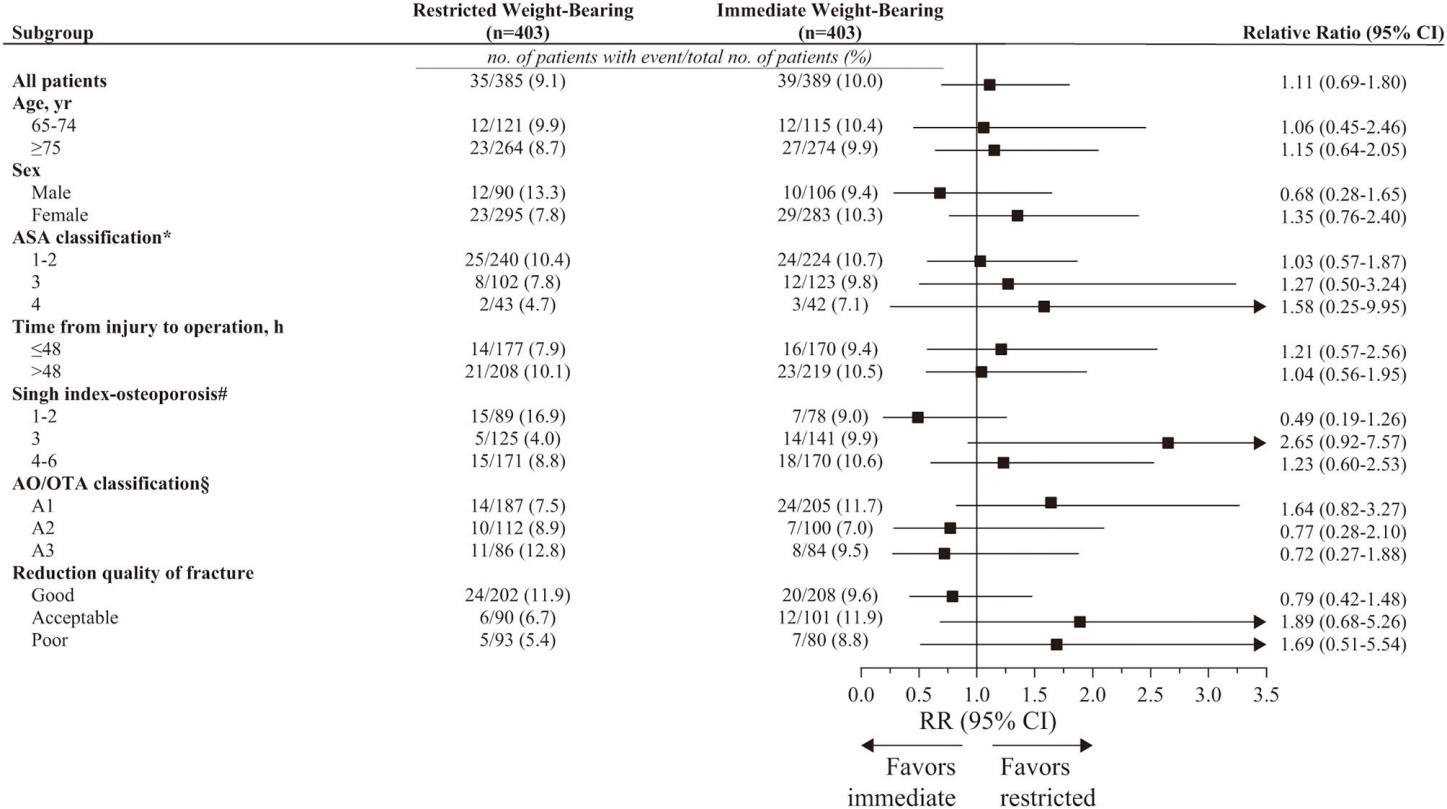
Prespecified subgroup analysis for implant failure at 12 months in PS-matched patients. CI, confidence intervals; AO/OTA, AO Foundation/Orthopedic Trauma Association; RR, relative risk. ^*^Range, 1 to 6; higher level indicates greater risk during anesthesia. Classifications include 1 (a healthy patient with no disease), 2 (a patient with mild systemic disease), 3 (a patient with severe systemic disease), 4 (a patient with severe systemic disease i.e., life-threatening), 5 (a patient who is not expected to survive with surgery), and 6 (a patient in whom brain death has occurred). ^#^Range, 1 to 6; lower level indicates more severe osteoporosis. Grade 1 (even the principal compressive trabeculae are markedly reduced in number and are no longer prominent), Grade 2 (only the principal compressive trabeculae stand out prominently; the others have been resorbed more or less completely), Grade 3 (there is a break in the continuity of the principal tensile trabeculae opposite the greater trochanter; this grade indicates definite osteoporosis), Grade 4 (principal tensile trabeculae are markedly reduced in number but can still be traced from the lateral cortex to the upper part of the femoral neck), Grade 5 (the structure of principal tensile and principal compressive trabeculae is accentuated. Ward's triangle appears prominent), and Grade 6 (all the normal trabecular groups are visible, and the upper end of the femur seems to be completely occupied by cancellous bone). ^§^Range, A1–A3; different classifications indicate different types of fracture. A1 (simple fracture), A2 (comminuted fracture involving the lateral cortex), and A3 (reverse oblique fracture).

For primary outcomes at 12-month follow-up, the subgroup analyses were consistent with the main findings (Figure 4). Results of sensitivity analyses using multiple imputation for implant failure at 12 months were consistent with the results of the primary analysis (9.9% [40/403 patients] in the restricted weight-bearing group vs. 10.2% [41/403 patients] in the immediate weight-bearing group; absolute risk difference, 0.25% [95% CI, −3.95% to 4.45%]; RR, 1.03 [95% CI, 0.65 to 1.63]; *p* = 0.91). As per the result of the stabilized IPTW, the outcome was consistent with the primary analysis (9.6% [39.9/415 patients] in the restricted weight-bearing group vs. 8.4% [45.3/540 patients] in the immediate weight-bearing group; absolute risk difference, −1.24% [95% CI, −5.06% to 2.39%]; RR, 0.86 [95% CI, 0.55 to 1.34]; *p* = 0.51). The sensitivity analysis, which focused on 145 young patients (aged 18 to 64 years), included 78 patients (53.8%) who underwent restricted weight-bearing and 67 patients (46.2%) who underwent immediate weight-bearing (Supplementary Table S2). As per the results of the outcomes, the results of younger patients were similar to the main findings (4.8% [3/62 patients] in the restricted weight-bearing group vs. 5.5% [4/73 patients] in the immediate weight-bearing group; absolute risk difference, 0.64% [95% CI, −7.07% to 8.35%]; RR, 1.21 [95% CI, 0.23 to 6.49]; *p* = 0.83). *E*-value for point estimate was 1.46, which meant the observed risk ratio of 1.11 could be explained away by an unmeasured confounder that was associated with both the treatment and the outcome by a risk of 1.46-fold each, above and beyond the measured confounders, but weaker confounding could not do so.

## Discussion

This retrospective cohort study involving older patients with intertrochanteric hip fractures showed that the receipt of immediate weight-bearing as tolerated, compared with restricted weight-bearing, did not increase the risks of postoperative implant failure (hip varus deformity, screw cutout, stress fracture of femoral shaft, and nonunion) or decrease the postoperative functional outcomes. Patients with immediate weight-bearing as tolerated after surgery were associated with a shorter time to full weight-bearing.

Previous studies have shown that restricted weight-bearing was associated with various adverse events, such as pneumonia, surgical site infections, blood transfusions, and delirium (13, 14, 31), which could be regarded as a supplement to this research. However, these studies did not evaluate the rate of fixation failure, which was the largest risk of early, unrestricted weight-bearing (11, 13, 14, 31). Therefore, the primary outcome of this study was the rate of fixation rate. Data from this article showed that no substantial difference was observed between patients with immediate weight-bearing as tolerated and those with restricted weight-bearing for postoperative implant failure at 3- and 12-month follow-up. As we know, about 2–3 weeks after surgery, soft callus was formed, which roughly corresponded to the time when the fragments were no longer moving freely. In addition, although patients receiving immediate weight-bearing as tolerated in this study were trained to start weight-bearing exercises as tolerated within 7 days after surgery, the standing and walking were accomplished with the assistive device. Meanwhile, the limitation on weight-bearing was only dependent on their perception of pain or swelling at the site of the fracture. The goal was not to achieve full weight-bearing as quickly as possible. Therefore, the rates of implant failure in the immediate weight-bearing group were equal to those in the restricted weight-bearing group.

Pfeufer et al. (32) found through gait analysis that weight-bearing restriction in elderly patients with hip fractures led to a loss of mobility. Although postoperative mobility was not investigated in this study, the functions of lower limbs and hips were evaluated. In addition, we found that there was no significant difference in postoperative functional outcomes between the 2 groups. It has been reported that several factors may affect the postoperative functional outcomes of intertrochanteric hip fractures, including bone quality, quality of fracture reduction, fracture classification, and implant selection (30). In this study, the above factors of patients were roughly similar between the 2 groups. Meanwhile, the fracture had healed during the 12-month follow-up if there was no accident. Therefore, the type of weight-bearing had little effect on the functional outcomes at this time. Furthermore, the rehabilitation process after surgery for two groups was the same.

This study had several limitations. First, when evaluating the primary outcome, appropriately 4% of patients lost to follow-up that was considered as the missing data. Thus, multiple imputations were used, and the main results were consistent with the primary analysis. Second, given its retrospective observation design, there was potential for unmeasured confounding and bias. Therefore, PS matching, subgroup analyses, and sensitivity analyses were used to assuage the confounding bias. In spite of these procedures, the possibility of residual confounding still existed, which may have influenced outcomes. Therefore, randomized studies are required to corroborate the present findings. Third, this single-center design may limit the generalizability of these results. Thus, multicenter, random, and large sample studies should be carried out to increase the credibility and generalizability of the study. Fourth, this study lacked a precise analysis of true weight-bearing. The difference between the two groups in the study was the time to start weight-bearing, and the rehabilitation process after starting weight-bearing was the same. Despite this, we will use mobile force sensors to detect the true weight-bearing in further studies. Fifth, we calculated *E*-value to evaluate the influence of residual confounding factors on the results and found that residual confounding factors did not overturn the conclusions of the primary analyses. Finally, except for intertrochanteric fractures, it was not clear whether there was any difference in the experience of other fractures among surgeons. This may have effects on the treatment of intertrochanteric hip fractures. However, PFNA-II technique is generally less invasive than other methods and is a relatively simple surgery to perform. Therefore, even if differences existed among surgeons regarding their experience with other types of fractures, the implications of our results may be minimal.

In conclusion, among older patients with intertrochanteric hip fractures, immediate weight-bearing as tolerated did not increase the risks of postoperative implant failure or worsen the functional outcomes compared with restricted weight-bearing. However, patients receiving immediate weight-bearing as tolerated had shorter time to full weight-bearing. These findings support the receipt of immediate weight-bearing as tolerated after surgery for older patients with intertrochanteric hip fracture and enable surgeons to make recommendations more confidently about optimal weight-bearing following fracture.

## Data Availability Statement

The original contributions presented in the study are included in the article/Supplementary Material, further inquiries can be directed to the corresponding author/s.

## Ethics Statement

This study was approved by the Institutional Board of East Hospital, Tongji University School of Medicine. Written informed consent for participation was not required for this study in accordance with the national legislation and the institutional requirements.

## Author Contributions

XJ conceptualized and designed the study, collected and analyzed the data, and wrote the manuscript. MQ conceptualized and designed the study and collected and analyzed the data. KZ conceptualized and designed the study, collected the data, and edited the manuscript. QH performed the operations and collected the data. YW collected and analyzed the data and wrote the manuscript. YC conceptualized and designed the study, collected and analyzed the data, performed the operations, wrote the manuscript, and supervised the study. All authors contributed to the article and approved the submitted version.

## Conflict of Interest

The authors declare that the research was conducted in the absence of any commercial or financial relationships that could be construed as a potential conflict of interest.

## Publisher's Note

All claims expressed in this article are solely those of the authors and do not necessarily represent those of their affiliated organizations, or those of the publisher, the editors and the reviewers. Any product that may be evaluated in this article, or claim that may be made by its manufacturer, is not guaranteed or endorsed by the publisher.
