# Supplementary material for: Influence of Timing of Postoperative Weight-Bearing on Implant Failure Rate Among Older Patients With Intertrochanteric Hip Fractures: A Propensity Score Matching Cohort Study
Source: Front Med (Lausanne). 2021 Dec 20;8:795595. doi: 10.3389/fmed.2021.795595 (PMC8720847; doi:10.3389/fmed.2021.795595)
Supplement: Supplementary file 1 [file Data_Sheet_1.docx]

***Supplementary Material***

**Influence of Timing of Postoperative Weight-Bearing on Implant Failure Rate Among Older Patients with Intertrochanteric Hip Fractures: A Propensity Score Matching Cohort Study**

**Supplementary Methods**

***E-Values for Evaluating Unmeasured Confounding in Observational Studies***

The E-value, introduced by VanderWeele and Ding in 2016 [1], was developed to evaluate how strong the unmeasured confounder (U) must be to negate observed results ($\mathrm{RR}_{\mathrm{ED}}^{\mathrm{true}}$, where RR is the risk ratio between exposure of interested (E) and the outcome (D)) in observational studies. When $\mathrm{RR}_{\mathrm{ED}}^{\mathrm{true}}$=1 (no association between E and D) and RR_EU_ =RR_UD_,

E-value = $\mathrm{RR}_{\mathrm{ED}}^{\mathrm{obs}}$ + $\sqrt{\mathrm{RR}_{\mathrm{ED}}^{\mathrm{obs}}\times(\mathrm{RR}_{\mathrm{ED}}^{\mathrm{obs}}-1)}$

Hence, the E-value is the minimum strength of association measured by a risk ratio scale that an unmeasured confounder would need to have with both the exposure and the outcome, after adjustment for the measured covariates, to fully explain away the specific exposure-outcome association. The E-value does not require any assumptions about unmeasured confounders; for example, that the unmeasured confounders are not necessarily binary, that more than 1 unmeasured confounder is present, and that there is no interaction between E, U, and D. An E-value can be calculated for unadjusted and adjusted odds ratios (OR), hazard ratios (HR), and risk differences (RD) after simple transformation of OR, HR, or RD to RR. For example, when the outcome is relatively rare (<15%) by the end of follow-up, the HR is similar to the RR. When the outcome is common (>15% at the end of follow-up), the RR = (1-0.5^sqrt(HR)^)/(1-0.5^sqrt(1/HR)^). An online E-value calculator (https://evalue.hmdc.harvard.edu/app/) is available to calculate E-values [2]. The E-value for an estimate, and for the limit of a 95%CI closest to the null, can be calculated in a straightforward way for risk ratios (Table A1) and for other measures [3].

**Table A1. Calculating the E-Value for Risk Ratios**

| **Estimate, by Direction of Risk Ratio** |  | Computation of the E-Value |
| --- | --- | --- |
| ***RR > 1*** |  |  |
| Estimate |  | E-value=RR + sqrt{RR × (RR −1)} |
| ***RR < 1*** |  |  |
| Estimate |  | Let RR* = 1/RR  E-value = RR* + sqrt{RR* × (RR*−1)} |

**Reference**

1. Ding P, VanderWeele TJ. Sensitivity Analysis Without Assumptions. *Epidemiology*. 2016;27:368-377.
2. Mathur MB, Ding P, Riddell CA, VanderWeele TJ. Web Site and R Package for Computing E-values. *Epidemiology*. 2018;29:e45-e47.
3. VanderWeele TJ, Ding P. Sensitivity Analysis in Observational Research: Introducing the E-Value. *Ann Intern Med*. 2017;167(4):268-274.

**Supplementary Figure 1.** **Directed Acyclic Graph (DAG) used to identify potential confounders**

**
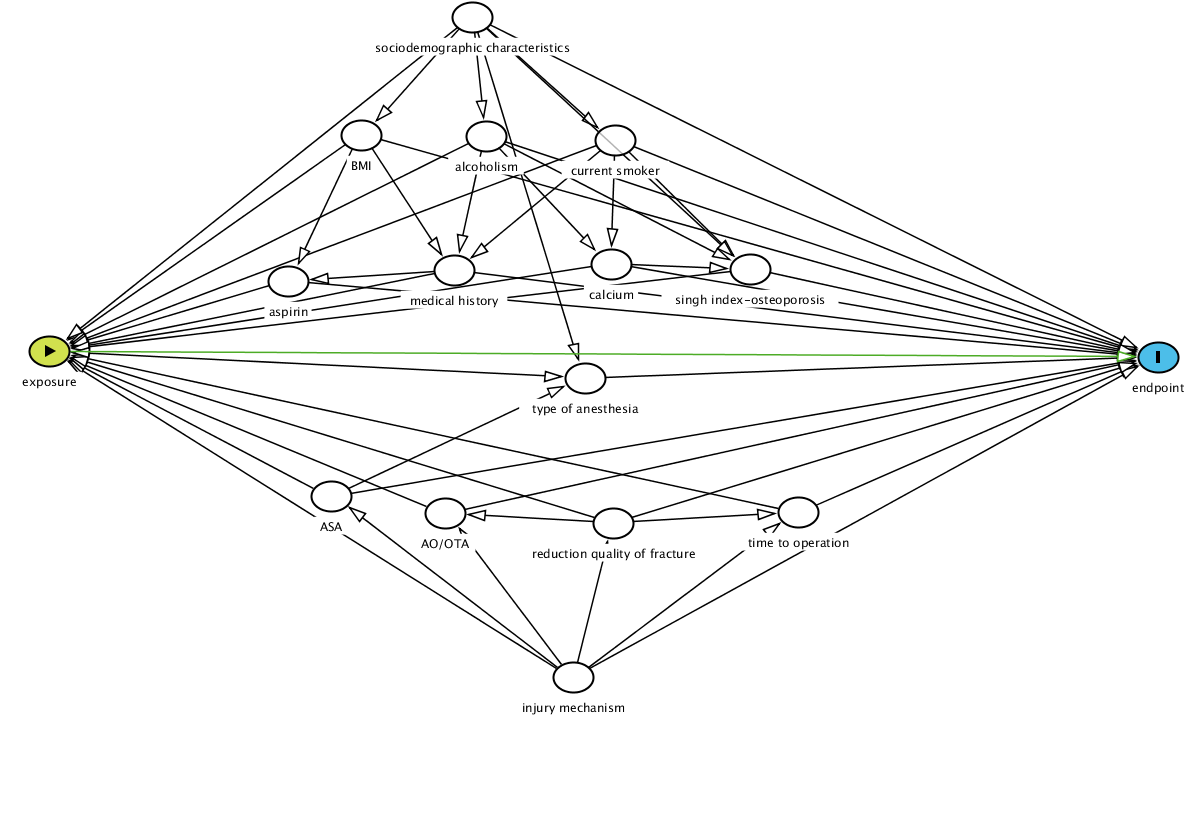
**

The DAG created using DADitty (*Textor J, et al. Int J Epidemiol. 2016*). White ovals represent potential confounders. Based on DAG, the minimal sufficient adjustment sets included sociodemographic characteristics (age, gender, geographic region, and educational level), BMI (body-mass index), ASA classification, injury mechanism, time to operation, Singh index-osteoporosis, AO/OTA classification, reduction quality of fracture, type of anesthesia, alcoholism, current smoker, aspirin and/or clopidogrel use, calcium and/or vitamin D use, and medical history for estimating the total effect of type of weight-bearing on endpoint.

**Supplementary Figure 2. Distribution of propensity score before and after propensity score matching in patients with immediate weight-bearing as tolerated or restricted weight-bearing**

**
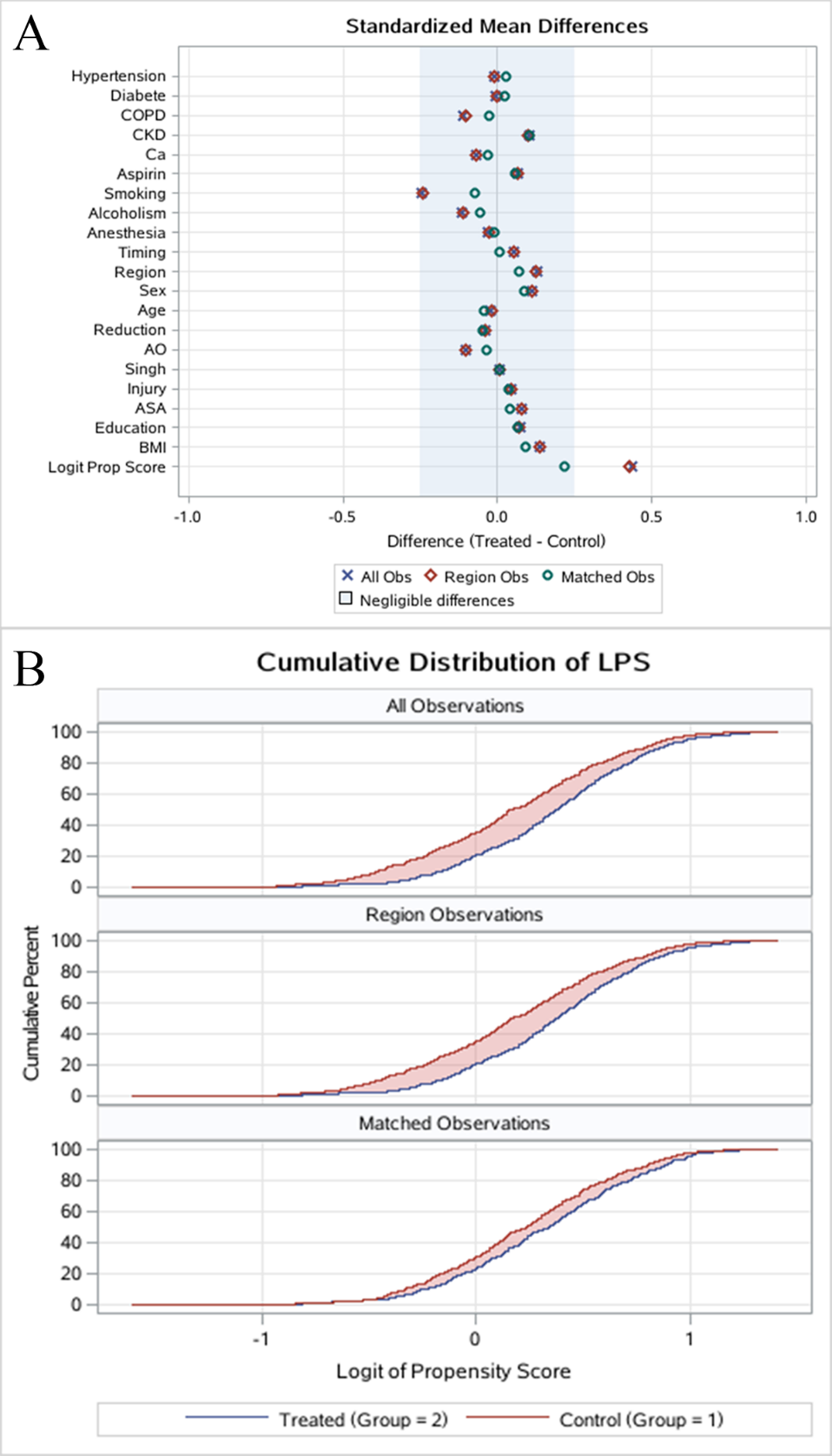
**

Abbreviation: COPD, chronic obstructive pulmonary disease; CKD, chronic kidney disease; Ca, calcium; AO, AO Foundation/Orthopaedic Trauma Association; BMI, body-mass index; LPS, logit propensity score.

The treatment group represented the immediate weight-bearing group. The control group represented the restricted weight-bearing group.

**Supplementary Table 1. Baseline demographic characteristic of younger patient (aged 18 to 64 years) with intertrochanteric hip fractures**

| **Demographics** |  | **Restricted Weight-Bearing**  **(n=78)*** |  | **Immediate Weight-Bearing**  **(n=67)*** |  | ***P* Value** |
| --- | --- | --- | --- | --- | --- | --- |
| **Age, y** |  |  |  |  |  | 0.91 |
| 18-44 |  | 24 (30.8) |  | 20 (29.9) |  |  |
| 45-64 |  | 54 (69.2) |  | 47 (70.1) |  |  |
| **Female** |  | 43 (55.1) |  | 33 (49.3) |  | 0.48 |
| **Body-mass index#** |  |  |  |  |  | 0.76 |
| <20 |  | 10 (12.8) |  | 11 (16.4) |  |  |
| 20-25 |  | 39 (50.0) |  | 30 (44.8) |  |  |
| >25 |  | 29 (37.2) |  | 26 (38.8) |  |  |
| **Geographic region** |  |  |  |  |  | 0.04 |
| Coastland |  | 45 (57.7) |  | 27 (40.3) |  |  |
| Inland |  | 33 (42.3) |  | 40 (59.7) |  |  |
| **Education level** |  |  |  |  |  | 0.59 |
| Primary school |  | 17 (21.8) |  | 14 (20.9) |  |  |
| Junior high school |  | 23 (29.5) |  | 25 (37.3) |  |  |
| Senior high school or above |  | 38 (48.7) |  | 28 (41.8) |  |  |
| **ASA classification§** |  |  |  |  |  | 0.57 |
| 1-2 |  | 53 (67.9) |  | 50 (74.6) |  |  |
| 3 |  | 17 (21.8) |  | 13 (19.4) |  |  |
| 4 |  | 8 (10.3) |  | 4 (6.0) |  |  |
| **Injury mechanism** |  |  |  |  |  | 0.20 |
| Falling from height |  | 37 (47.4) |  | 22 (32.8) |  |  |
| Traffic accident |  | 31 (39.7) |  | 34 (50.7) |  |  |
| Other |  | 10 (12.8) |  | 11 (16.4) |  |  |
| **Time from injury to operation, h** |  |  |  |  |  | 0.09 |
| ≤48 |  | 31 (39.7) |  | 36 (53.7) |  |  |
| >48 |  | 47 (60.3) |  | 31 (46.3) |  |  |
| **Singh index-osteoporosis¶** |  |  |  |  |  | 0.79 |
| 1-2 |  | 15 (19.2) |  | 16 (23.9) |  |  |
| 3 |  | 28 (35.9) |  | 22 (32.8) |  |  |
| 4-6 |  | 35 (44.9) |  | 29 (43.3) |  |  |
| **AO/OTA classification†** |  |  |  |  |  | 0.37 |
| A1 |  | 31 (39.7) |  | 25 (37.3) |  |  |
| A2 |  | 27 (34.6) |  | 30 (44.8) |  |  |
| A3 |  | 20 (25.6) |  | 12 (17.9) |  |  |
| **Reduction quality of fracture** |  |  |  |  |  | 0.64 |
| Good |  | 46 (59.0) |  | 40 (59.7) |  |  |
| Acceptable |  | 20 (25.6) |  | 20 (29.9) |  |  |
| Poor |  | 12 (15.4) |  | 7 (10.4) |  |  |
| **Type of anesthesia** |  |  |  |  |  | 0.38 |
| General |  | 73 (93.6) |  | 60 (89.6) |  |  |
| Spinal or epidural |  | 5 (6.4) |  | 7 (10.4) |  |  |
| **Alcoholism** |  | 10 (12.8) |  | 5 (7.5) |  | 0.29 |
| **Current smoker** |  | 25 (32.1) |  | 18 (27) |  | 0.50 |
| **Aspirin and/or clopidogrel use** |  | 3 (3.8) |  | 3 (4.5) |  | 0.59 |
| **Calcium and/or vitamin D use** |  | 4 (5.1) |  | 5 (7.5) |  | 0.41 |
| **Medical history** |  |  |  |  |  |  |
| Chronic kidney disease |  | 3 (3.8) |  | 4 (6.0) |  | 0.55 |
| COPD |  | 3 (3.8) |  | 2 (3.0) |  | 0.78 |
| Diabetes |  | 10 (12.8) |  | 13 (19.4) |  | 0.28 |
| Hypertension |  | 41 (52.6) |  | 45 (67.2) |  | 0.07 |

Abbreviations: ASA, American Society of Anesthesiologists; AO/OTA, AO Foundation/Orthopaedic Trauma Association; COPD, chronic obstructive pulmonary disease.

* Data are expressed as number (percentage) of patients unless otherwise indicated; Percentages may not total 100 because of rounding.

# The body-mass index is the weight in kilogram divided by the square of the height in meters.

§ Range, 1 to 6; higher level indicates greater risk during anesthesia. Classifications include 1 (a healthy patient with no disease), 2 (a patient with mild systemic disease), 3 (a patient with severe systemic disease), 4 (a patient with severe systemic disease that is life-threatening), 5 (a patient who is not expected to survive with surgery), and 6 (a patient in whom brain death has occurred).

¶ Range, 1 to 6; lower level indicates more severe osteoporosis. Grade 1 (even the principal compressive trabeculae are markedly reduced in number and are no longer prominent), Grade 2 (only the principal compressive trabeculae stand out prominently; the others have been resorbed more or less completely), Grade 3 (there is a break in the continuity of the principal tensile trabeculae opposite the greater trochanter; this grade indicates definite osteoporosis), Grade 4 (principal tensile trabeculae are markedly reduced in number but can still be traced from the lateral cortex to the upper part of the femoral neck), Grade 5 (the structure of principal tensile and principal compressive trabeculae is accentuated. Ward’s triangle appears prominent), and Grade 6 (all the normal trabecular groups are visible, and the upper end of the femur seems to be completely occupied by cancellous bone).

† Range, A1 to A3; different classification indicates different type of fracture. A1 (simple fracture), A2 (comminuted fracture involving the lateral cortex), and A3 (reverse oblique fracture).
